# Male age mediates reproductive investment and response to paternity assurance

**DOI:** 10.1098/rspb.2013.1124

**Published:** 2013-08-07

**Authors:** Kyle M. Benowitz, Megan L. Head, Camellia A. Williams, Allen J. Moore, Nick J. Royle

**Affiliations:** 1 Centre for Ecology and Conservation, College of Life and Environmental Sciences, University of Exeter, Cornwall Campus, Penryn TR10 9EZ, UK; 2 Department of Genetics, University of Georgia, Athens, GA 30602, USA

**Keywords:** burying beetle, mating effort, parental care, parental selection, terminal investment, paternity

## Abstract

Theory predicts that male response to reduced paternity will depend on male state and interactions between the sexes. If there is little chance of reproducing again, then males should invest heavily in current offspring, regardless of their share in paternity. We tested this by manipulating male age and paternity assurance in the burying beetle *Nicrophorus vespilloides*. We found older males invested more in both mating effort and parental effort than younger males. Furthermore, male age, a component of male state, mediated male response to perceived paternity. Older males provided more prenatal care, whereas younger males provided less prenatal care, when perceived paternity was low. Adjustments in male care, however, did not influence selection acting indirectly on parents, through offspring performance. This is because females adjusted their care in response to the age of their partner, providing less care when paired with older males than younger males. As a result offspring, performance did not differ between treatments. Our study shows, for the first time, that a male state variable is an important modifier of paternity–parental care trade-offs and highlights the importance of social interactions between males and females during care in determining male response to perceived paternity.

## 1. Introduction

Life-history theory predicts that parents who provide care for their offspring should balance investment in current and future reproduction in such a way that maximizes lifetime reproductive success [1–4]. Thus, any factor that decreases the benefits or increases the costs of caring for the current brood should reduce a parent's investment in the current reproductive attempt in favour of future reproduction [5,6]. In species with paternal care, this means that fathers with a low share in paternity, owing to female use of stored sperm [7], female participation in extra-pair copulations [8] or reproductive contributions by satellite or sneaker males [9] should lead to decreased levels of paternal care [10–14]. Empirical evidence for a relationship between paternity and paternal care, however, is equivocal. Recently, it has been suggested that this lack of support for a relationship between parentage and parental effort stems from a tendency to treat paternity as a property of a species or individual male, rather than from an emergent property of interactions within and between the sexes [15–17]. Consequently, relationships between parentage and parental care can be understood only by considering interactions between males and females and variation among individuals in state [17,18].

When individuals vary in their potential for future reproduction owing to differences in condition, they are also expected to differ in how they respond to variation in paternity. Males with high potential for future reproduction are predicted to respond to reduced paternity by being more likely to desert the current brood to seek further mating opportunities elsewhere [19]. By contrast, males with little potential for future reproduction are expected to invest more effort in the current brood, regardless of their share in paternity, because they are unlikely to reproduce again [20].

Another factor that may influence the correlation between parental care and paternity is parental selection [21]. Parental selection is indirect selection acting on parent phenotypes owing to their effects on offspring fitness. Because offspring carry the genes for adult traits, selection on earlier stages influences the distribution of these genes in the population before the trait is expressed. Thus, if reduced paternal effort decreases offspring performance, then selection acting on males through their related offspring will limit the evolution of male response to paternity assurance. If, however, females compensate for adjustments in male care [22], then parental selection on males will be relaxed, because fitness differences among the young will be minimized. In order to examine how the potential for future reproduction mediates male behavioural responses to variation in certainty of paternity, we manipulated a component of male state through manipulating male age, and paternity assurance through manipulating the perception of potential competitors in the environment, then quantified offspring performance traits in a the burying beetle *Nicrophorus vespilloides*. Ageing is a major component of state [23]. Burying beetles are well suited to testing hypotheses relating paternal effort to variation in perceived paternity. They provide biparental care to their offspring, and compete for and breed on the carcasses of small vertebrates, which are an essential, but unpredictable resource [24]. Individuals compete with other members of their sex on the carcass, and the dominant male and female form a pair that defends and prepares this resource for larvae [25]. Females and males mate both on and off a carcass, and females that arrive on a carcass often carry stored sperm, so multiple paternity of broods is common [26,27].

Parental care in burying beetles can be split into prenatal and postnatal phases of caring [28]. During prenatal care, males and females prepare and defend the carcass from other beetles of the same sex, but still engage in mating with members of the opposite sex. Males also defend paternity through mate guarding and repeated mating [26,29]. During postnatal care, both males and females can provide direct care to offspring in the form of provisioning regurgitated food as well as indirect care in the form of continued carcass maintenance and defence [25,30]. Parental effort is highly variable in this species and either parent may desert the carcass before larvae complete development, although males tend to desert earlier than females [25,27,31]. In the wild, *N. vespilloides* experience variable sex ratios throughout the season (P. Hopwood and N. J. Royal 2012, unpublished data), suggesting that individuals will experience variation in certainty of paternity throughout their lifetime [20].

Key empirical and theoretical work shows that in order to understand relationships between parentage and parental care, it is necessary to take account of future expected mating success and paternity (e.g. owing to variation in age) and the response of females to adjustments in effort by their partners [17,20]. Here, we provide such a test. We tested two predictions regarding behavioural adjustments of mating and paternal effort. First, we predicted that older males would invest more in the current reproductive attempt than younger males because they have reduced potential for future reproduction (*sensu* [1]). Second, we predicted that if potential for future reproduction is important in determining how males respond to certainty of paternity, then younger males would be more responsive to potential competitors in the environment that might affect paternity (reduce care if paternity assurance is low), whereas older males would be more likely to maintain high levels of care, regardless of their perceived paternity assurance [20]. Finally, we also assessed the potential for parental selection to act on adjustment of male behaviour by examining interactions between male and female behaviour and the effects of male age and paternity assurance on offspring performance. If adjustments in parental care reduce offspring performance, then selection acting indirectly on parents (parental selection) may limit the evolution of male adjustment of care in response to either male age or paternity assurance [21].

## 2. Methods

All beetles used in this experiment were from recently established laboratory stock. Our stock population originated from 80 male and 80 female *N. vespilloides,* caught in Devichoys Wood, Cornwall, UK (N 50^o^11′ 47″ E 5^o^7′23″) between May and August 2011, and was maintained as a randomly mated outbred population. Details on stock maintenance are given in reference [32]. Prior to use in this experiment, we isolated larvae from randomly paired individuals and reared them in small plastic containers (7 × 7 × 4 cm) filled with 2 cm of damp soil in an incubator (16 L : 8 D, 21 ± 1°C). Once larvae became pupae, they were checked once a day to determine the date they eclosed to adulthood. Upon eclosion, we determined the sex of adults and began to feed them two decapitated mealworms (*Tenebrio*) twice a week.

### (a) Experimental design

We used a 2 × 2 factorial design to test the effects of male age and paternity assurance on parental care behaviour and offspring fitness. For logistical reasons, our experiment was conducted over four blocks that differed only in the date they were set up. There was no effect of block on any of our analyses and so it was not considered further.

Upon eclosion, males were allocated to one of two age treatments: younger males (between 11 and 14 days old post-eclosion) and older males (between 35 and 38 days old post-eclosion). In our laboratory, even in the absence of predators and competition for food, beetles can live for 10 weeks but substantial mortality begins to occur from fourth to fifth weeks of age (P. Hopwood and N. J. Royal 2012, unpublished data). Prior to the experiment, all males were kept in identical conditions except that soil in the containers of older beetles was replaced every two weeks to avoid accumulation of mites, which are highly successful in laboratory conditions [33]. All males were socially naive prior to the experiments (i.e. reared alone).

Prior to breeding, males were assigned to one of two paternity treatments: either low or high perceived likelihood of paternity based on the previous presence of a male, which we term ‘paternity assurance’. For both treatments, a freshly thawed mouse carcass was placed in a breeding box (i.e. a transparent plastic container: 17 × 12 × 6 cm) with 1 cm of soil. We used mice only between 20 and 25 g (weighed to 0.01 gm) to control for size of breeding resource, as carcass size is an important determinant of offspring size [34]. In our low paternity assurance treatment, we placed a ‘competitor’ male in the breeding box with the mouse (all competitor males were between 11 and 14 days old). After 20 h, competitor males were removed, and a female was added to each breeding box. Females were allowed to acclimate for four hours prior to focal males being added to the breeding box and the commencement of mating trials. The expectation is that females and males can detect the previous presence of the male via odour. Burying beetle behaviour is strongly influenced by odour, and males and females can detect categories of other individuals through their cuticular hydrocarbons [35–37]. Our high paternity assurance treatment differed in that no male beetle was added to the breeding box prior to the addition of females, and so there were no odours of other males. This manipulation of paternity assurance was found to alter male behaviour in preliminary experiments (C. A. Williams and N. J. Royal 2010, unpublished data), and allowed us to alter the perception of the presence of competitors without altering mating experience of females, which is desirable for avoiding differences in female behaviour owing to mating [18].

### (b) Mating effort

We observed male and female beetles in their breeding boxes immediately after the addition of the focal male. There was only a thin layer of soil, so all mating could be observed. We scan sampled each breeding box every minute for 20 min. Mean mating duration in this species is 90 s [38]. Each pair was given a score of 1 if they were observed in copula or 0 if they were not. We then calculated mating effort as the total number of observations in which the pair was found mating. This score captures variation in both the number of times the pair mate and the duration of each mating. There is no obvious courtship behaviour in *N. vespilloides*. Variation in the duration and frequency of mating results from male persistence and female resistance [38]. Previous studies have shown that mating frequency of a pair decreases with time (over 5 h [38] and over 24 h [39]), however, this decrease is consistent across contexts suggesting that any differences in mating frequency found between our treatments over the 20 min observation period used here is likely to be representative of longer-term patterns in mating behaviour. In addition, House *et al*. [38] measured mating behaviour over short observation periods (50 min) and found that both male mating duration and male mating frequency are heritable in this species [38]. This further indicates that measuring mating behaviour over short-time periods is suitable for detecting between male variation in mating behaviour.

### (c) Parental effort: prenatal care

Prenatal parental care in burying beetles involves preparation and maintenance of the food resource for eventual offspring, and can be measured as the proportion of time that parents are observed on the carcass performing care behaviours [24,25]. This includes stripping the fur and skin from the carcass, burying the resource and forming it into a ball. The parents also smear anal secretions on the carcass to prevent bacterial growth and manipulate the carcass with their mouthparts to prevent fungal growth [40,41] and guard the carcass from inter- and intraspecific competitors [24]. During this period, male beetles also perform behaviours to ensure paternity by repeatedly mating and mate guarding (by remaining near or on the female) [27,38]. Mate guarding involves the male riding on the back of the female or following close behind her. Parental care is associated with attendance to the carcass [24,25] and is easily distinguished from resting or self-grooming, and mating and mate guarding [30,34,42–44].

After mating trials, male and female pairs were placed in an incubator (16 L : 8 D, 21 ± 1°C). The next morning (10–12 h later), we began checking individual breeding boxes twice daily (approx. every 12 h) to determine whether the female and male were present or absent from the carcass. Beetles present on the carcass were observed performing prenatal care. Our observations of prenatal care continued until larvae were added to the carcass (between 72 and 144 h after the male was added to the breeding box, the normal time of larval arrival [45]), giving a total number of six to 12 prenatal care observations per beetle.

During observations of prenatal care, we also checked breeding boxes to determine the onset of egg laying. Eggs can easily be seen in the soil through the bottom of the clear plastic breeding box. Female egg laying usually begins around 20 h after a pair is placed on a carcass and continues for 24–60 h [45]. We found that eggs begin hatching around 60 h after being laid. For this reason, we removed the parents and carcass from the original breeding box to an identical box at 48 h after the onset of egg laying, thus isolating parents from their larvae before hatching. We continued to check boxes containing eggs twice per day for hatching larvae.

### (d) Parental effort: duration of postnatal care

Our measure of postnatal parental care was duration of care, which was the most appropriate available measure, because time on the carcass is time that could be spent seeking further reproductive opportunities elsewhere. We controlled the number of larvae arriving at the carcass and cross-fostered, so that all parents cared for larvae unrelated to both parents as well as to each other [28]. Once larvae hatched, they were counted, pooled in a separate container and given a piece of ground beef to eat. We kept track of when larvae hatched and family membership, so that we could ensure that adult pairs received 20 unrelated larvae within 24 h of when their own larvae hatched. This allowed us to ensure that differences in parental care and offspring fitness were not influenced by larval number or coevolved parent–offspring behaviour [42], and controlled for variation in parental care and offspring size and development that occurs owing to variation in brood number [34,43].

We measured duration of postnatal care by determining how long parents remained with the larvae. Postnatal care in burying beetles begins when larvae arrive on the carcass after hatching [45] and involves both direct care (regurgitation of food to larvae, predigesting food for larvae) and indirect care (maintenance and defence of the carcass) [24,25,30]. Parents also engage in non-parental behaviour such as grooming and resting. These are performed on or near the carcass. Males and females do not mate after larvae arrive. To measure duration of postnatal care, we recorded when a parent abandoned the carcass. After larvae were added to the carcass, we checked twice per day and recorded the location of the adults. To determine accurately the location of adults in the breeding container, it was necessary to lift the carcass and disturb the soil close to the carcass. While such disturbance may lead beetles to shorten their duration of care (M. L. Head 2012, unpublished data), any such effects will be random with respect to treatment, and there is no reason to suggest this leads to bias in our results. Individuals were recorded as having abandoned the brood when they were observed far away from the carcass for two consecutive observations. Beetles that have abandoned broods do not subsequently return to feed young. The robustness of our definition of abandonment of broods was confirmed through observations of male beetles in the wild using motion-detection cameras (P. Hopwood and N. J. Royal 2013, unpublished data).

### (e) Offspring performance

We measured three offspring performance traits associated with offspring fitness and potentially influenced by parental care [42]: survival, mass and development. The number of larvae that survived was counted at dispersal. We calculated offspring mass by collecting and weighing all larvae from each brood at dispersal and dividing by the number of larvae to obtain the average larval mass for each family. Once larvae disperse (which they do together), they do not feed again until they are adult. We also calculated the time spent from birth to dispersal as a measure of development. Larval development on the carcass varies for each family [42] and lasts between 88 and 120 h. By using family values for each of our offspring performance measures, we avoid pseudo-replication and inflated degrees of freedom.

### (f) Statistical analysis

To investigate how male age and paternity assurance influenced male mating effort, male and female parental effort and offspring performance, we used generalized linear models (GLMs) with appropriate error structures. Response variables were transformed using the power transform function in the R package CAR. All models included male age and paternity assurance treatment as fixed effects as well as the interaction between them. All GLMs were conducted using R v. 2.13.1 [46]. Sample size was 118 pairs for all parental effort analyses (young/low perceived paternity = 26, young/high perceived paternity = 28, old/low perceived paternity = 30, old/high perceived paternity = 34).

Finally, we conducted path analysis to determine the relationships between the traits measured in more detail. Path analysis allowed us to determine the relative strength of both direct and indirect pathways. The model used in our path analysis was determined *a priori* using information from previously published research [44]. We report standardized path coefficients, because male duration of care and female duration of care are both dependent and predictor variables [47]. Path analysis was conducted using the SPSS add on AMOS v. 20 module. Data have been deposited in the Dryad repository.

## 3. Results

### (a) Mating effort

**Figure 1.**
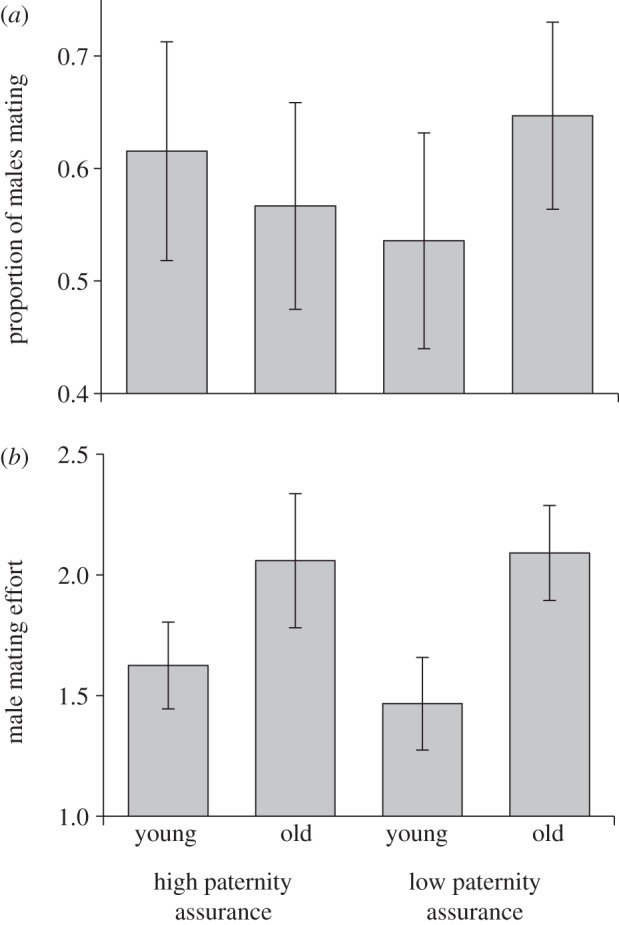
Effects of male age and paternity assurance on mating behaviour (mean ± s.e.m.). (*a*) The proportion of males that mated (*n* = 118). (*b*) The number of observations males were observed in copula (*n* = 70).

There was no statistically significant effect of male age, paternity assurance or the interaction between them on the likelihood of males mating at least once (GLM with quasi-binomial errors, *n* = 118; male age, *F*_1,116_ = 0.149, *p* = 0.703; paternity assurance, *F*_1,115_ = 0.006, *p* = 0.939; interaction, *F*_1,114_ = 0.751, *p* = 0.388; figure 1*a*). For males that copulated at least once, however, male age had a significant effect on mating effort measured as the number observations males were observed in copula. Older males were observed mating more often than younger males (GLM with quasi-Poisson errors, *n* = 70; *F*_1,68_ = 6.101, *p* = 0.016). Neither paternity assurance treatment nor the interaction between male age and paternity assurance influenced male mating effort (paternity assurance, *F*_1,67_ = 0.061, *p* = 0.806; interaction, *F*_1,66_ = 0.239, *p* = 0.627; figure 1*b*).

### (b) Parental effort: prenatal care

**Figure 2.**
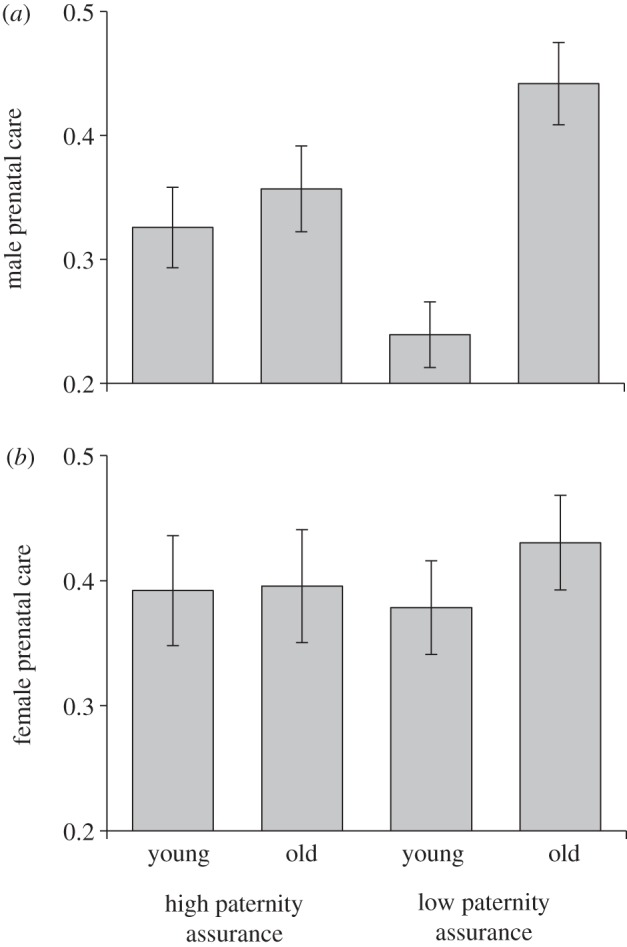
The effects of male age and paternity assurance on prenatal care (mean ± s.e.m.). (*a*) The proportion of prenatal observations that males were observed on the carcass (*n* = 118). (*b*) The proportion of prenatal observations that females were observed on the carcass (*n* = 118).

The amount of time that males spent on the carcass prior to the arrival of larvae was significantly affected by the interaction between male age and paternity assurance treatment (*F*_1,114_ = 6.538, *p* = 0.012). When assurance of paternity was high, both younger and older males spent an intermediate amount of time preparing the carcass. However, older males increased their amount of time spent on prenatal care when paternity was uncertain, whereas younger males decreased care (figure 2*a*). There was also a main effect of male age, with older males providing more prenatal care (*F*_1,116_ = 10.985, *p* = 0.001). There was no main effect of paternity treatment (*F*_1,115_ = 0.073, *p* = 0.788), on male prenatal care.

The amount of time that females spent on prenatal care was not affected by male age, paternity assurance or the interaction between them (male age, *F*_1,116_ = 0.709, *p* = 0.401; paternity assurance, *F*_1,115_ = 0.012, *p* = 0.913; interaction, *F*_1,114_ = 0.422, *p* = 0.517; figure 2*b*).

### (c) Parental effort: postnatal care

**Figure 3.**
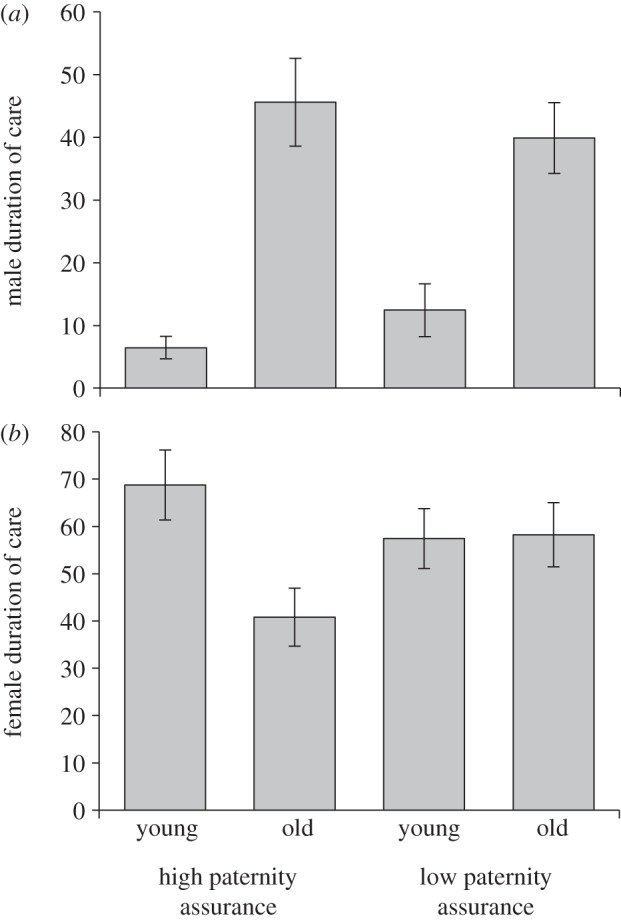
Effects of male age and paternity assurance on postnatal care (mean ± s.e.m.). (*a*) Duration of male care (hours; *n* = 118). (*b*) Duration of female care (hours; *n* = 118).

Male age had a significant effect on the duration of care provided by males (*F*_1,116_ = 45.734, *p* < 0.001). Older males remained on the carcass longer than younger males (figure 3*a*). There was no significant effect of paternity assurance on male duration of care (*F*_1,115_ = 0.005, *p* = 0.944) nor was there an interaction between male age and paternity assurance (*F*_1,114_ = 2.239, *p* = 0.137).

**Figure 4.**
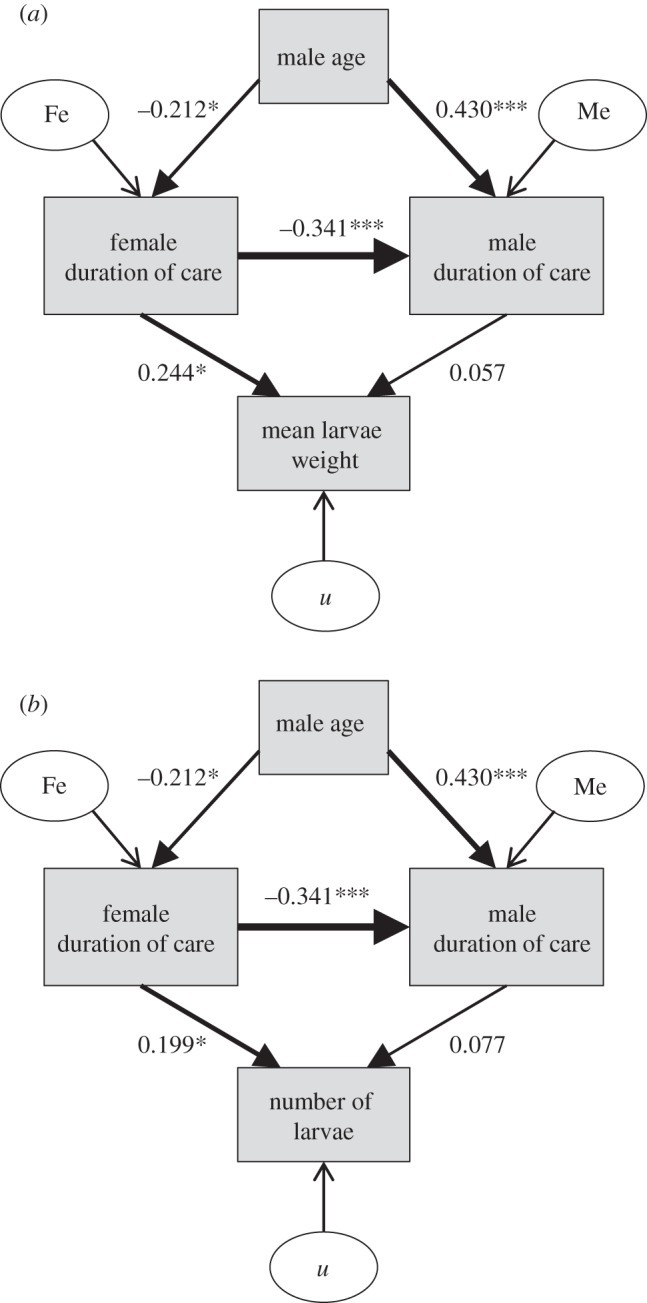
Path analysis showing direct and indirect effects of male age on male and female duration of care and how these influence offspring performance. The variable *u* stands for residual, Fe for female error and Me for male error. (*a*) Paths affecting mean larvae weight (*n* = 118). (*b*) Paths affecting number of larvae surviving (*n* = 118, *p < 0.05, **p < 0.01, ***p < 0.001).

In contrast to results found for prenatal care, female postnatal care was influenced by male age (*F*_1,116_ = 4.226, *p* = 0.042). Overall females cared longer when paired to younger males, however, this pattern appears to be mostly driven by females in our high paternity assurance treatment (figure 3*b*), as indicated by the marginally non-significant interaction effect of male age and paternity assurance (*F*_1,114_ = 3.836, *p* = 0.053). The main effect of paternity assurance (*F*_1,115_ = 0.952, *p* = 0.331), on the other hand, had no effect on the duration of female postnatal care. Path analysis confirmed this pattern, revealing that male duration of care is influenced both by a direct effect of male age in addition to indirect effects on female duration of care. This means that older males care for longer not only because they are old, but also because females provide less care when paired with older males (figure 4).

### (d) Offspring performance

**Figure 5.**
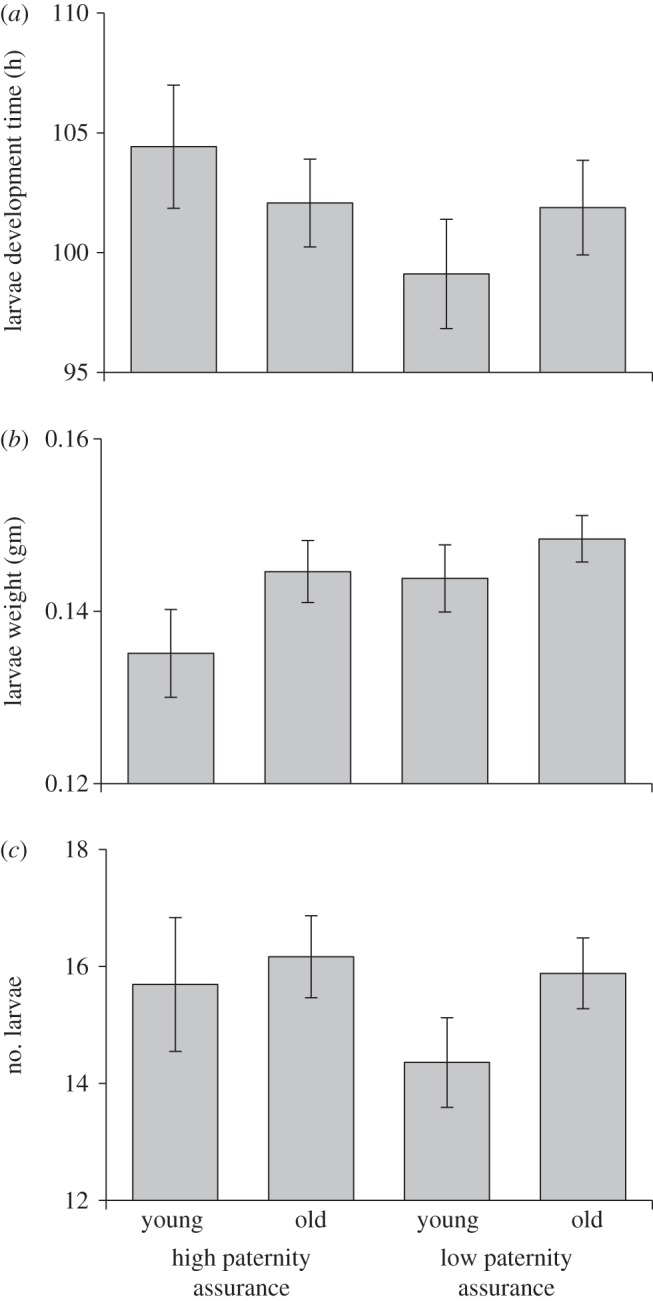
Effects of male age and paternity assurance on offspring performance (mean ± s.e.m.). (*a*) Effects on time to offspring dispersal from the carcass (*n* = 119). (*b*) Effects on average offspring mass (*n* = 118). (*c*) Effects on number of offspring surviving to dispersal (*n* = 118).

There was no statistically significant effect of male age, paternity assurance or the interaction between them on average offspring development time to dispersal (male age, *F*_1,116_ = 0.019, *p* = 0.889; paternity assurance, *F*_1,115_ = 1.390, *p* = 0.241; interaction, *F*_1,114_ = 1.413, *p* = 0.237; figure 5*a*), offspring mass (male age, *F*_1,116_ = 2.497, *p* = 0.117; paternity assurance, *F*_1,115_ = 2.067, *p* = 0.153; interaction, *F*_1,114_ = 0.301, *p* = 0.585; figure 5*b*) or offspring survival (male age, *F*_1,116_ = 1.158, *p* = 0.284; paternity assurance, *F*_1,115_ = 1.393, *p* = 0.240; interaction, *F*_1,114_ = 0.696, *p* = 0.406; figure 5*c*). Path analysis showed that the lack of effect of male age on offspring performance measures is most likely due to the negative relationship between the duration of male and female care (figure 4), resulting in equivalent overall effects of parental care on offspring, regardless of variation in age or paternity assurance. These patterns are found, regardless of which measurement of offspring performance is considered (figure 4*a,b*).

## 4. Discussion

In species with paternal care, fathers are expected to balance investment in current and future reproduction to maximize their lifetime reproductive success [1–4]. Theoretical work indicates that male investment in current offspring will depend on a male's potential for future reproduction and his expected share of paternity in the current brood [20], with male adjustment of parental effort also expected to depend on male state [17,18]. We therefore predicted that individuals with greater potential for future reproduction would invest less in current reproduction and be more responsive to changes in perceived paternity than individuals with a low probability of future reproduction. We found that older males invested more in current mating and parental effort than younger males, as predicted. We also found that male age-mediated male response to paternity assurance, with younger males decreasing and older males increasing prenatal care in response to decreased paternity assurance. Despite reduced parental effort of younger males (and particularly those uncertain of their paternity), we found no evidence this decreased effort reduced offspring performance. Path analysis suggests this lack of parental selection on paternal effort may have occurred because females adjusted their parental effort based on the age of their mate. Selection acting indirectly on parents through offspring performance is therefore unlikely to constrain the evolution of behavioural plasticity in parental effort by males in relation to their age or their confidence in the paternity of their current brood. Our results highlight the importance of the state of individuals and social interactions between males and females in determining the relationship between parentage and parental effort [17].

Older males, with lower potential for future reproduction than younger males, invested more time in postnatal parental effort. This result is similar to those found previously in some avian species [48,49]. The terminal investment hypothesis predicts that for organisms where the likelihood of mortality increases with age, older individuals should invest more in reproduction than younger individuals [50]. Alternatively, if there is differential survival of high-quality individuals that also provide better care, this can result in a pattern of greater care in older individuals without age-dependent changes in reproductive investment [49,51,52]. Finally, if parents learn how to care for offspring or acquire resources, experience may play an important role in how much parents are able to invest in their offspring [53]. The increased investment towards offspring by older males in the current study is most consistent with the terminal investment hypothesis; older males in our experiment had no prior breeding experience and mortality in our laboratory population between the ages used in our experiment (two to five weeks post-eclosion) is negligible.

Female burying beetles also show terminal investment in postnatal care [28,54,55]. In female burying beetles, prenatal investment in offspring declines with age at first reproduction [28,56]. This decline is due to a combination of female restraint and senescence [56]. However, older females compensate for lower prenatal investment during postnatal care by increasing provisioning rates [28]. The similar patterns of reproductive investment by males and females across their lifetime is not surprising as the potential for future reproduction are unlikely to differ markedly between the sexes in this species. In burying beetles, the scarcity and unpredictability of resources required for breeding is likely to mean that older individuals have little chance of future reproduction regardless of their sex, selecting for terminal investment in both males and females. Despite the expectation that males and females are likely to have different age-dependent reproduction strategies even in species with biparental care [57], few studies have compared differences in how male and female parental effort changes with age within a single species (but see reference [31]).

Our study manipulated male age to explicitly test the hypothesis that males with high potential for future reproduction should reduce parental effort in response to decreased paternity, whereas males with low potential for future reproduction should maintain high levels of care that result from the increased value of the current breeding attempt. We found that males adjusted prenatal care in response to varying levels of paternity assurance, but that this response interacted with male age. Older males increased prenatal care, whereas younger males decreased prenatal care in response to low paternity assurance. This result supports predictions that male response to paternity assurance is likely to be state-dependent and in particular to depend on a male's potential for future reproduction [18,20].

The influence of male age on the relationship between perceived paternity and parental care was apparent only during prenatal care. The lack of an interaction between age and perceived paternity on the duration of postnatal care may be because the effects of male age override any effects of the paternity assurance treatment. Even when perceived paternity is high, younger males abandoned early, whereas older males provided care for much longer. Further response to paternity treatment may therefore be limited. Alternatively, there may be important differences in the costs and benefits to males of providing prenatal versus postnatal care. One important difference between prenatal and postnatal care in burying beetles is the potential for males to influence paternity in the current reproductive attempt. During prenatal care, males and females continue to mate while preparing the carcass and male-repeated mating is an important predictor of paternity [26]. By staying close by the female males may also be able to prevent competitors from mating with the female [39]. This may increase the benefits to males of providing prenatal care [58].

If the ability to influence paternity is a hidden benefit of prenatal care, then why do younger males decrease their care in response to uncertain paternity rather than increasing care (as old males do) and ensuring paternity? One possible explanation is suggested by our data on mating effort. Younger males were observed mating less often than older males. This may indicate a female preference for older mates, or lower investment by younger males in mating. If the difference in mating rate between younger and older males, we observed continued throughout prenatal care, then it might have provided males with further cues of their likelihood of siring offspring in the current reproductive attempt. Such cues may contribute to the pattern that we see here of decreased prenatal care for young males in response to uncertain paternity and increased prenatal care by older males reflecting higher paternity assurance. In species with biparental care, the level of care that males and females provide offspring may depend on the level of care provided by their partner [22]. Females may respond either directly to variation in male state or indirectly via behavioural differences resulting from variation in male state. In *N. vespilloides*, females do not generally adjust parental care behaviour in response to variation in male behaviour [5,44]. This suggests that females actively altered levels of parental effort in terms of duration of care in direct response to male age rather than to behaviour. By contrast, male burying beetles do adjust parental care in response to variation in female care behaviour [44,59]. As such, older males increase their duration of care as a result of terminal investment, but also in response to decreased care by their partner.

Increased duration of care by older males means that females paired with these males can achieve equal reproductive success to females paired with younger males, but with less effort. Parental care is completed before larvae disperse [24,30], and males staying longer may shorten the duration females have to stay. Shorter duration of female care allows females to return to the breeding pool sooner as well as conserve resources for future reproduction [60]. These benefits for females of mating with older males may provide strong selection for female mating preferences based on male age. Finally, the interaction that we see between male and female care also has important implications for the evolution of plasticity in paternal care. If paternal care is important for offspring fitness, lowering male effort should result in reduced offspring performance and parental selection (indirect selection through offspring) should counter altered parental care behaviour. We found that younger males with higher future reproductive potential invested less in current reproduction. However, females cared more when mated to younger males, so there is unlikely to be parental selection against male adjustment of parental effort. Such compensatory adjustments by parents to changes in effort by their partners have also been shown in many other species and are important for the stability of biparental care under sexual conflict [22]. Our results indicate that indirect selection on parents does not limit the evolution of male adjustment of effort in response to differences in age or paternity assurance, which may help explain the stability of biparental care in this species.

In conclusion, we show that male burying beetles respond to variation in both the potential for future reproduction and paternity assurance by adjusting parental effort. The evolution of this response depends not only on the behaviour of the male, but also on interactions with his partner, which affect offspring performance. Our study therefore demonstrates the importance of studying the relationship between parentage and parental effort by taking into account both variation in state of the individuals involved and the social environment in which the behaviours are expressed [15,16].

## Data accessibility

Data has been deposited in the Dryad repository. http://dx.doi.org/10.5061/dryad.n022p.
